# Alignment of brain embeddings and artificial contextual embeddings in natural language points to common geometric patterns

**DOI:** 10.1038/s41467-024-46631-y

**Published:** 2024-03-30

**Authors:** Ariel Goldstein, Avigail Grinstein-Dabush, Mariano Schain, Haocheng Wang, Zhuoqiao Hong, Bobbi Aubrey, Samuel A. Nastase, Zaid Zada, Eric Ham, Amir Feder, Harshvardhan Gazula, Eliav Buchnik, Werner Doyle, Sasha Devore, Patricia Dugan, Roi Reichart, Daniel Friedman, Michael Brenner, Avinatan Hassidim, Orrin Devinsky, Adeen Flinker, Uri Hasson

**Affiliations:** 1 Business School, Data Science department and Cognitive Department, Hebrew University, Jerusalem, Israel; 2 Google Research, Tel Aviv, Israel; 3 Department of Psychology and the Neuroscience Institute, Princeton University, Princeton, NJ USA; 4 New York University Grossman School of Medicine, New York, NY USA; 5 Faculty of Industrial Engineering and Management, Technion, Israel Institute of Technology, Haifa, Israel; 6 School of Engineering and Applied Science, Harvard University, Cambridge, MA USA; 7 New York University Tandon School of Engineering, Brooklyn, NY USA

**Keywords:** Neural encoding, Neural decoding, Language

## Abstract

Contextual embeddings, derived from deep language models (DLMs), provide a continuous vectorial representation of language. This embedding space differs fundamentally from the symbolic representations posited by traditional psycholinguistics. We hypothesize that language areas in the human brain, similar to DLMs, rely on a continuous embedding space to represent language. To test this hypothesis, we densely record the neural activity patterns in the inferior frontal gyrus (IFG) of three participants using dense intracranial arrays while they listened to a 30-minute podcast. From these fine-grained spatiotemporal neural recordings, we derive a continuous vectorial representation for each word (i.e., a brain embedding) in each patient. Using stringent zero-shot mapping we demonstrate that brain embeddings in the IFG and the DLM contextual embedding space have common geometric patterns. The common geometric patterns allow us to predict the brain embedding in IFG of a given left-out word based solely on its geometrical relationship to other non-overlapping words in the podcast. Furthermore, we show that contextual embeddings capture the geometry of IFG embeddings better than static word embeddings. The continuous brain embedding space exposes a vector-based neural code for natural language processing in the human brain.

## Abstract (web summary)

Here, using neural activity patterns in the inferior frontal gyrus and large language modeling embeddings, the authors provide evidence for a common neural code for language processing.

## Introduction

Deep language models (DLMs) trained on massive corpora of natural text provide a radically different framework for how language is represented in the brain. The recent success of DLMs in modeling natural language can be traced to the gradual development of three foundational ideas in computational linguistics.

The first key innovation was to (1) embed words in continuous vector space: Traditionally, words in language were viewed as discrete symbolic units in a lexicon^1,2^. Early work in distributional semantics demonstrated that the meaning of words could instead be captured by geometric relationships in a continuous vector space based on co-occurrence statistics in large corpora of text^3^. More recent models—for example, GloVe^4^—factorize co-occurrence matrices into a semantically rich embedding space. This vector-space representation of linguistic structure would prove to be a core component of DLMs. The second key innovation was to (2) use self-supervised neural networks to embed the statistical structure of natural language: Rather than curating an embedding space from a precalculated co-occurrence matrix or using easily-interpretable, hand-crafted features, researchers are leveraging the power of self-supervised neural networks to learn the embedding space directly from natural language. For example, the neural network model word2vec^5^ embeds a complex semantic structure in the embedding space based on a simple self-supervised objective combined with nonlinear transformations between layers. The scalability of this approach would prove to be transformative for DLMs. The third key innovation was to (3) leverage the local relationships among words to contextualize the embedding space: The previous generation of models, such as GloVe and word2vec, assign a single static embedding to each word capturing the global meaning of that word across all contexts. This approach, however, does not do justice to the subtle, context-sensitive dependencies across words in natural language. DLMs—like GPT-2^6^—have capitalized on the transformer architecture^7^ to assign each word in a text a unique, context-sensitive meaning based on the surrounding words within a given context window. The contextual embeddings learned by DLMs capture subtle statistical dependencies reflecting syntactic, semantic, and pragmatic relationships among words^8–10^. The synthesis of these three innovations—using self-supervised deep learning to encode the context-sensitive statistical structure of natural language in a continuous embedding space—has culminated in full-fledged language models capable of generating novel sentences with human-like, context-sensitive linguistic structure.

Similar computational principles were found essential for understanding the neural basis of language processing in the human brain. The first key principle was to shift toward (1) distributed, vector-space neural codes: Traditionally, neuroscience was focused on understanding the functional properties of single neurons and individual brain areas. In the last decades, there has been a gradual shift toward distributed, vector-space representation, such as neural population codes^11–14^, multivoxel pattern analysis^15–17^, and representational geometry^18–20^. Interestingly, early examples of this work^11^ roughly coincide with developing fundamental ideas about distributed representation in neural networks^21^. Groundbreaking work by Mitchell and colleagues^22^ begins to synthesize these parallel developments by using co-occurrence-based semantic vectors (similar to GloVe embeddings) to predict multivoxel patterns of brain activity associated with single words (see also ^23,24^). The second key principle emerged with the adoption of (2) deep statistical-learning models as a computational framework for how the brain processes natural stimuli: Researchers began to move away from simplistic experimental paradigms to model the multidimensional neural responses in real-life contexts^25,26^, positioning deep neural networks as a computational framework for neural processing of naturalistic visual and auditory stimuli^27–33^. The extension of this approach to language coincides with the third key principle of (3) using contextual embeddings to understand context-sensitive language processing in the brain: With the recent development of context-sensitive DLMs, we see an explosion of work demonstrating that contextual embeddings better predict brain activity than static embeddings^34–41^. These developments have revealed several computational principles the brain shares with DLMs: the brain incorporates prior context into the meaning of individual words^34,38,40^, spontaneously predicts forthcoming words^40,42^, and computes post-word-onset prediction error signals^40,43^.

We provide two pieces of evidence to support this shift from a rule-based symbolic framework to a vector-based neural code for processing natural language in the human brain. First, we demonstrate that the patterns of neural responses (i.e., brain embeddings) for single words within a high-level language area, the inferior frontal gyrus (IFG), capture the statistical structure of natural language. Using a dense array of micro- and macro-electrodes, we sampled neural activity patterns at a fine spatiotemporal scale that has been largely inaccessible to prior work relying on fMRI and EEG/MEG. This allows us to directly compare the representational geometries of IFG brain embeddings and DLM contextual embeddings with unprecedented precision. A common definition of ‘geometry’ is a branch of mathematics that deals with shape, size, the relative position of figures, and the properties of shapes^44^. This and other work in cognitive computational neuroscience rely on a long history of work in psychology, neuroscience, and machine learning to characterize the geometric relationships among vector-based representations in a multidimensional space; for example, the geometric relationships among stimuli in an internal psychological space^45–47^; the geometric relationships among patterns of brain activity^11,17,18^; and the geometric relationships among embeddings in a neural network^21,29^.

Second, one of the core commitments emerging from these developments is that DLMs and the human brain have common geometric patterns for embedding the statistical structure of natural language^32^. But how precise is this mapping? In the current work, we build on the zero-shot mapping strategy developed by Mitchell and colleagues^22^ to demonstrate that the brain represents words using a continuous (non-discrete) contextual-embedding space. Unlike discrete symbols, in a continuous representational space, there is a gradual transition among word embeddings, which allows for generalization via interpolation among concepts. Using the zero-shot analysis, we can predict (interpolate) the brain embedding of left-out words in IFG based solely on their geometric relationships *to other words in the story*. We also find that DLM contextual embeddings allow us to triangulate brain embeddings more precisely than static, non-contextual word embeddings similar to those used by Mitchell and colleagues^22^. Together, these findings reveal a neural population code in IFG for embedding the contextual structure of natural language.

## Results

**Fig. 1:**
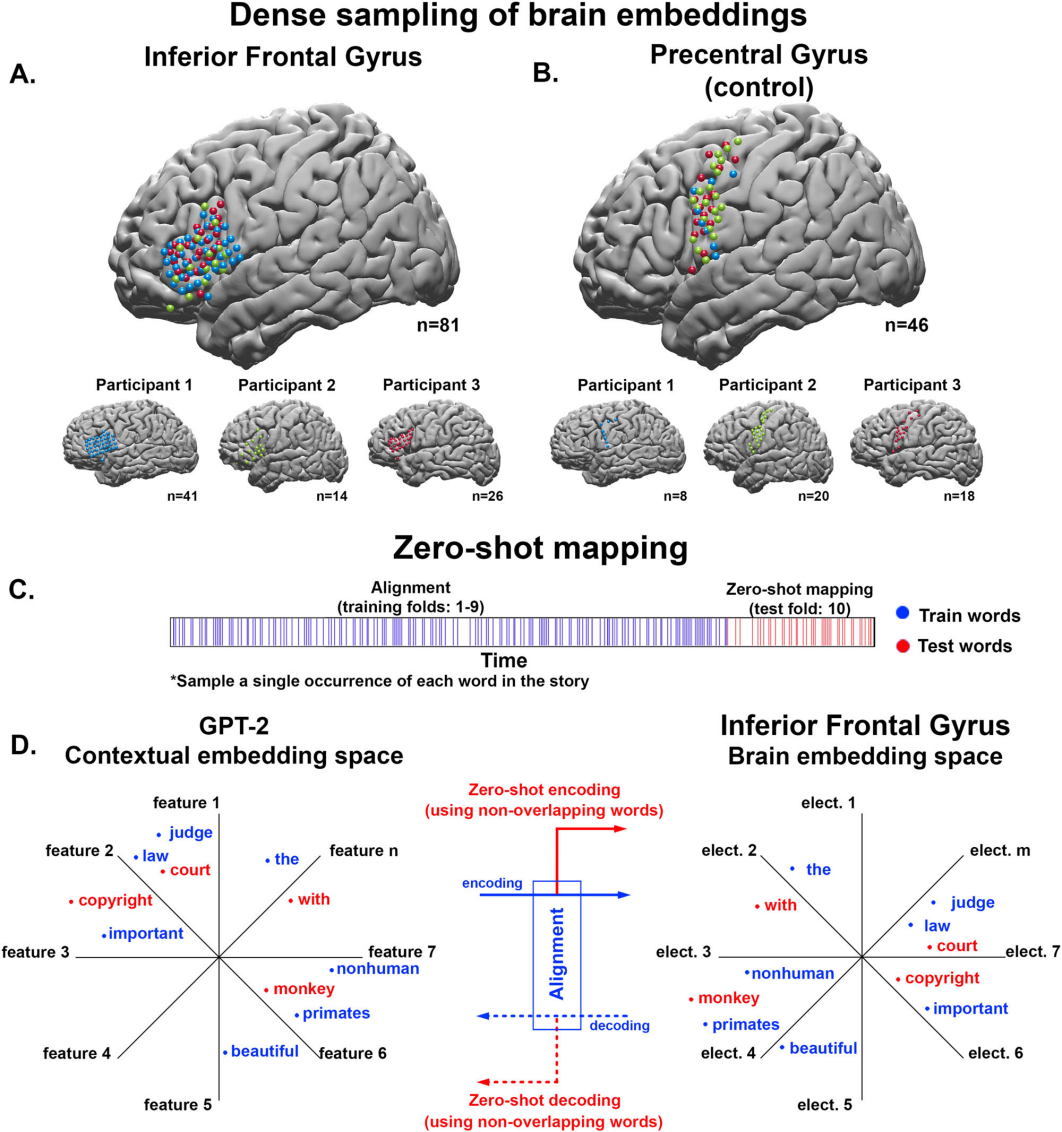
Zero-shot encoding and decoding analysis. **A** Dense coverage of the inferior frontal gyrus (IFG). Using the Desikan atlas^69^ we identified electrodes in the left IFG and precentral gyrus (pCG). **B** The dense sampling of activity in the adjacent pCG is used as a control area. **C** We randomly chose one instance for each unique word in the podcast (each blue line represents a word from the training set, and red lines represent words from the test set). This resulted in 1100 unique words, which we split into ten folds. Nine folds were used for training (blue), and one fold containing 110 unique, nonoverlapping words was used for testing (red). **D** left- We extracted the contextual embeddings from GPT-2 for each of the words. Using PCA, we reduced the contextual embeddings to 50 features. Right- We used the dense sampling of activity patterns across electrodes in IFG to estimate a brain embedding for each of the 1100 words. The brain embeddings were extracted for each participant and across participants. Center- We used nine training folds to estimate a model (e.g., using linear regression in the case of the encoding analysis), effectively aligning the GPT-2 contextual embeddings and the brain embeddings (multi-electrode activity) for each word in the training set. We then evaluate the quality of this alignment by predicting embeddings for test words not used in fitting the regression model; successful prediction is possible if there exists some common geometric patterns. Tfhe solid blue arrow denotes the alignment phase in which we align the contextual embeddings to the brain embeddings based on the training words; the solid red arrow denotes the evaluation phase of the encoding analysis, where we predict brain embeddings for novel words from the contextual embeddings. The dotted blue arrow denotes the alignment procedure of the decoding analysis, in which we align the brain embeddings to the contextual embeddings based on the training words; the dotted red arrow denotes the evaluation phase of the decoding analysis, where we predict contextual embeddings for novel words from the brain embeddings.

Using a dense array of electrocorticographic (ECoG) micro-and macro-electrodes, we recorded neural activity in the IFG of three epileptic participants while they listened to a 30-min audio podcast (see Materials and Methods). We focus on IFG as it has been positioned as a central hub for language processing, emphasizing semantic and syntactic processing^40,48–53^. Overall, we had a dense sampling of 81 intracranial electrodes in IFG, with 41, 14, and 26 electrodes in participants 1–3, respectively (Fig. 1A). The ECoG recordings provide a measure of the neural activity patterns for each word in the IFG. These activity patterns correspond to a local brain embedding for each word, where the activity of each electrode serves as a feature (i.e., dimension) in the IFG embedding space (Fig. 1D, left). Across all participants, we extracted an 81-dimensional brain embedding vector for each word in the story. The brain embeddings were sampled across all electrodes in IFG and identified anatomically during surgery without imposing any additional selection criteria. To assess the selectivity of the results, we also sampled activity from two anatomically adjacent brain regions containing a similar density of electrodes but not thought to be directly involved in language comprehension: the precentral gyrus and the postcentral gyrus.

We used zero-shot mapping, a stringent generalization test, to demonstrate that IFG brain embeddings have common geometric patterns with contextual embeddings derived from a high-performing DLM (GPT-2). The zero-shot analysis imposes a strict separation between the words used for aligning the brain embeddings and contextual embeddings (Fig. 1D, blue) and the words used for evaluating the mapping (Fig. 1D, red). We randomly chose one instance of each unique word (type) in the podcast, resulting in 1100 words (Fig. 1C). As an illustration, in case the word “monkey” is mentioned 50 times in the narrative, we only selected one of these instances (tokens) at random for the analysis. Each of those 1100 unique words is represented by a 1600-dimensional contextual embedding extracted from the final layer of GPT-2. The contextual embeddings were reduced to 50-dimensional vectors using PCA (Materials and Methods). We then divided these 1100 words’ instances into ten contiguous folds, with 110 unique words in each fold. Crucially, there was no overlap between the words in each fold. As an illustration, the chosen instance of the word “monkey” can appear in only one of the ten folds. We used nine folds to align the brain embeddings derived from IFG with the 50-dimensional contextual embeddings derived from GPT-2 (Fig. 1D, blue words). The alignment between the contextual and brain embeddings was done separately for each lag (at 200 ms resolution; see Materials and Methods) within an 8-second window (4 s before and 4 s after the onset of each word, where lag 0 is word onset). The remaining words in the nonoverlapping test fold were used to evaluate the zero-shot mapping (Fig. 1D, red words). Zero-shot encoding tests the ability of the model to interpolate (or predict) IFG’s unseen brain embeddings from GPT-2’s contextual embeddings. Zero-shot decoding reverses the procedure and tests the ability of the model to interpolate (or predict) unseen contextual embedding of GPT-2 from IFG’s brain embeddings.

### Zero-shot encoding

In the zero-shot encoding analysis, we use the geometry of the embedding space to predict (interpolate) the neural responses of unique words not seen during training. Specifically, we used nine folds of the data (990 unique words) to learn a linear transformation between the contextual embeddings from GPT-2 and the brain embeddings in IFG. Next, we used the tenth fold to predict (interpolate) IFG brain embeddings for a new set of 110 unique words to which the encoding model was never exposed. The test fold was taken from a contiguous time section and the training folds were either fully contiguous (for the first and last test folds; Fig. 1C) and split into two contiguous sections when the test folds were in the middle. Predicting the neural activity for unseen words forces the encoding model to rely solely on geometrical relationships among words within the embedding space. For example, we used the words “important”, “law”, “judge”, “nonhuman”, etc, to align the contextual embedding space to the brain embedding space. Using the alignment model (encoding model), we next predicted the brain embeddings for a new set of words “copyright”, “court”, and “monkey”, etc. Accurately predicting IFG brain embeddings for the unseen words is viable only if the geometry of the brain embedding space matches the geometry of the contextual embedding space. If there are no common geometric patterns among the brain embeddings and contextual embeddings, learning to map one set of words cannot accurately predict the neural activity for a new, nonoverlapping set of words.

**Fig. 2:**
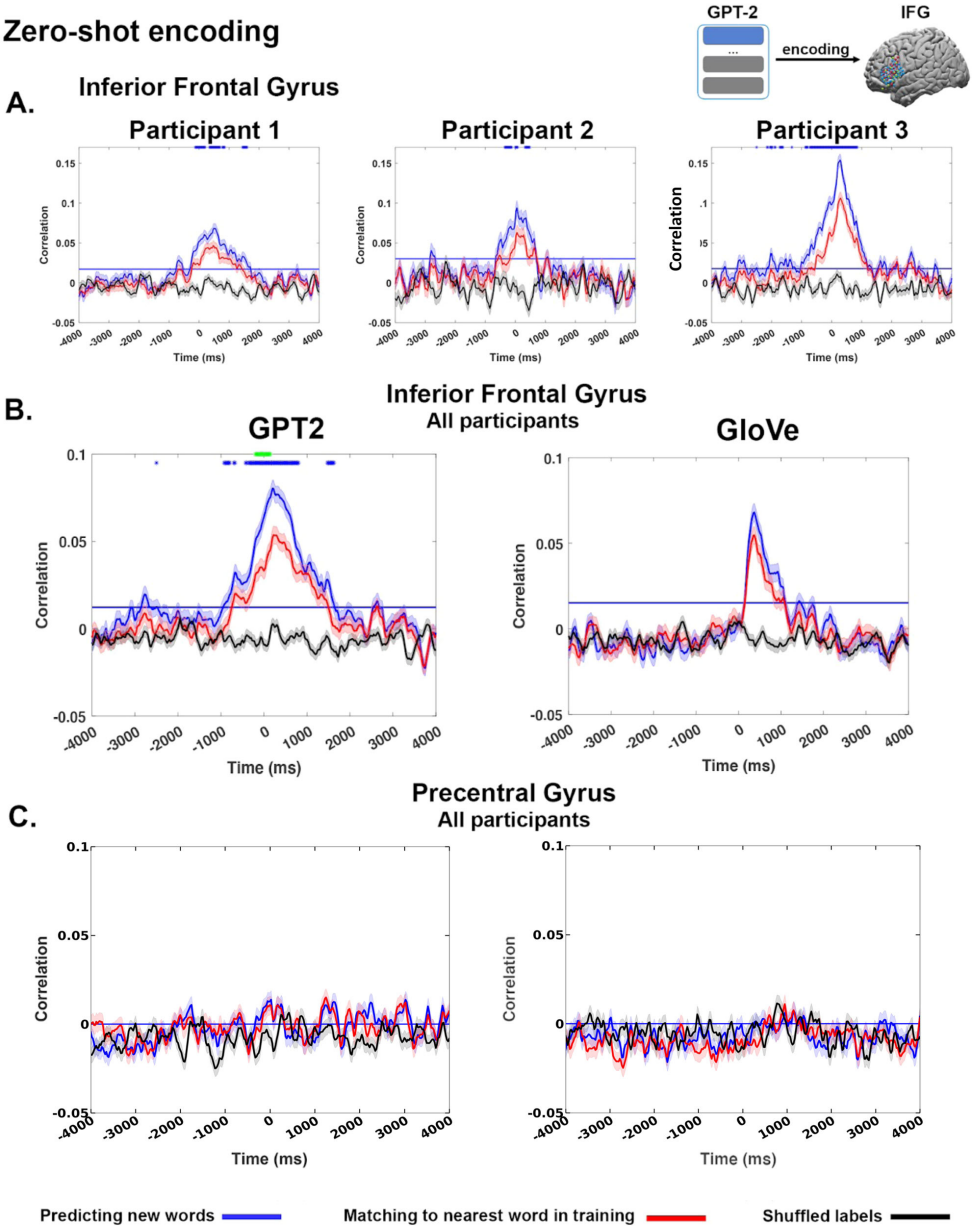
Encoding analysis reveals common geometric patterns between contextual embeddings and brain embeddings. **A** Zero-shot encoding between the contextual and brain embeddings in IFG for each patient. The solid blue line shows the average correlation between the predicted and actual brain embeddings in IFG for all words across all test sets (all shaded lines represent standard error above and standard error below average). Significant correlations peak after word onset but precede word onset (the significance threshold is marked by the horizontal blue line). The red line shows the zero-shot encoding for the word from the training set that is most similar (nearest neighbor) to each test word (error bands mark the standard error across words). Note that the reduced correlations for the nearest training embeddings indicate that the zero-shot mapping can accurately interpolate to new embeddings not seen during the training phase (at the level of individual patients). The blue asterisks represent a significant difference (one-sided, FDR corrected, *q* < 0.01) between the correlation with the actual contextual embeddings (blue line) and the correlation with the nearest embedding from the training set (red line). The black line shows the zero-shot encoding between shuffled contextual embeddings and the brain embeddings. **B** Zero-shot encoding for brain embeddings was extracted across all participants. The green asterisks (left) indicate significantly greater performance for GPT-2 embeddings versus GloVe embeddings (one-sided, FDR corrected, *q* < 0.01). All shaded lines represent standard error above and standard error below average. **C** Zero-shot encoding for electrodes sampled from the anatomically adjacent control area, the precentral gyrus. We did not find a significant correlation between brain embeddings and contextual embeddings observed in this non-linguistic area. All shaded lines represent standard error above and standard error below average.

In the zero-shot encoding analysis, we successfully predicted brain embeddings in IFG for words not seen during training (Fig. 2A, blue lines) using contextual embeddings extracted from GPT-2. We correlated the predicted brain embeddings with the actual brain embedding in the test fold. We averaged the correlations across words in the test fold (separately for each lag). The averaged correlations of the unseen words were significant at multiple time points surrounding word onset for all three participants (a blue horizontal line marks the significance threshold; for details on the significance test, see Materials and Methods), with peak correlations of roughly 200 ms after word onset. Furthermore, the encoding performance for unseen words was significant up to −700 ms before word onset, which provides evidence for the engagement of IFG in context-based next-word prediction^40^. The zero-shot mapping results were robust in each individual participant and the group level (Fig. 2B-left, blue lines).

For GPT-2 embeddings, the zero-shot encoding performance was close to zero when we randomly matched the words in the test fold with mismatching contextual embeddings (Figs. 2A, B and S1, black lines). Furthermore, the predicted neural activity pattern (brain embedding) for each unseen word was selective to the IFG. We did not find a statistically significant correlation between brain embedding and contextual embedding in an adjacent control area, the precentral gyrus (Fig. 2C, blue lines). However, using a one-sided nonparametric permutation test, we found a significant difference between the correlations in the IFG and in the precentral gyrus (*p* < 0.001). To ensure that the lack of zero-shot mapping in the precentral gyrus is not due to the lower spatial sampling, we replicated the findings in individual participants who have a comparable number of electrodes in the precentral gyrus and IFG (Fig. S1). We combined electrodes in precentral and postcentral gyri to increase coverage, but we did not find statistically significant effects of zero-shot mapping in control areas (Fig. S2). However, using a one-sided nonparametric permutation test, we found a significant difference between the correlations in the IFG and in the control area (*p* < 0.001).

### Precise neural interpolation based on common geometric patterns

The zero-shot encoding analysis suggests that the common geometric patterns of contextual embeddings and brain embeddings in IFG is sufficient to predict the neural activation patterns for unseen words. A possible confound, however, is the intrinsic co-similarities among word representations in both spaces. For example, the embedding for the test word “monkey” may be similar to the embedding for another word from the training set, such as “baboon” (in most contexts); it is also likely that the activation patterns for these words in the IFG are similar^22,24^.

To address this, we devised a control analysis to determine whether the zero-shot mapping can precisely predict the brain embedding of unseen words (i.e., left-out test words) relying on the common geometric patterns across both embedding spaces. We repeated the zero-shot mapping analysis—however, instead of using the actual contextual embedding for each of the unseen words in the test fold in predicting their brain embedding, we used the contextual embedding of the most similar word in the training set (based on the cosine similarity of the embeddings). If the nearest word from the training set yields similar performance, then the model predictions are not very precise and could simply be the result of memorizing the training set. However, if the prediction matches the actual test word better than the nearest training word, this suggests that the prediction is more precise and not simply a result of memorizing the training set. For example, instead of using the contextual embedding for “monkey” (a previously unseen word in the test fold), we used the contextual embedding of the most similar word in the training set (“baboon”; the similarity was computed using the cosine-similarity metric of the word embeddings). If the zero-shot analysis matches the predicted brain embedding with the nearest similar contextual embedding in the training set, switching to the nearest training embedding will not deteriorate the results. In contrast, if the alignment exposes common geometric patterns in the two embedding spaces, using the embedding for the nearest training word will significantly reduce the zero-shot encoding performance.

We observed a significant decrease in performance when using the nearest training embeddings (Fig. 2A, B left, red line). This is evident both at the group level and in each individual participant. This further supports the claim that alignment between the contextual and brain embeddings revealed fine-grained common geometric patterns between the two representational spaces (blue asterisk represents a significant difference (*p* < 0.01; for details, see Materials and Methods); that is, the predicted embedding for a given word more closely matches that word than even the most similar word from the training set.

To compute the contextual embedding for a given word, we initially supplied all preceding words to GPT-2 and extracted the activity of the last hidden layer (see Materials and Methods), ignoring the cross-validation folds. To rule out the possibility that our results stem from the fact that the embeddings of the words in the test fold may inherit contextual information from the training fold, we developed an alternative way to extract contextual embeddings. To ensure no contextual information leakage across folds, we first split the data into ten folds (corresponding to the test sets) for cross-validation and extracted the contextual embeddings separately within each fold. Specifically, (a) we sampled the unique words (1–1100); (b) we split them into contiguous folds of 110 words; (c) the first word in each fold was the first word supplied to GPT-2 (d) the last (i.e., 110th) word in each segment was the last word supplied to GPT-2; (e) we used these folds to extract embeddings. In this more strict cross-validation scheme, the word embeddings do not contain any information from other folds. We repeated the encoding and decoding analyses and obtained qualitatively similar results (e.g., Figs. S3–9). We also examine an alternative way to extract the contextual word embedding by including the word itself when extracting the embedding, the results qualitatively replicated for these embeddings as well (Fig. S4).

### Interpolation based on word embeddings versus contextual embeddings

Inspired by Mitchell and colleagues^22^, we performed the exact same zero-shot analysis using co-occurrence-based, static (GloVe) word embeddings. Replicating their results, we could interpolate the activity to unseen words using the geometry of the GloVe embedding (blue line, Fig. 2B right). However, performance was significantly weaker than for the contextual embeddings (*p* < 0.001; marked by green asterisks; FDR corrected). Furthermore, there was no statistical difference between the zero-shot analysis and the nearest neighbor control for the GloVe embeddings (Fig. 2C blue versus red lines). While there are common geometric patterns between the static GloVe embedding space and the IFG embeddings—sufficient for predicting the meaning of unseen words, as demonstrated by Mitchell and colleagues (Fig. 2B left blue line)—the alignment is not precise enough to interpolate the unique contextual meaning of test words better than related words from the training set (Fig. 2B left red line and Fig. S5).

The analysis report in Fig. 2 is very conservative, as the nearest neighbor is taken from the training set. This is a conservative analysis because the model is estimated from the training set, so it overfits the training set by definition. Even though it is trained on the training set, the model prediction better matches the brain embedding of the unseen words in the test than the nearest word from the training set. Thus, we conclude that the contextual embeddings have common geometric patterns with the brain embeddings. However, if we adopt less conservative criteria, inspired by Mitchell and colleagues, where the nearest neighbor word (i.e., not target word) was another word from the test set (meaning was not seen during the training set), we do get the effect for the GloVe (and a larger effect for the contextual embeddings; Fig. S5). We also controlled for the possibility that the effect results from merely including information from previous words. For this, we curated pseudo-contextual embeddings (not induced by GPT-2) by concatenating the GloVe embeddings of the ten previous words to the word in the test set and replicated the analysis (Fig. S6).

### Symbolic embeddings versus contextual (GPT-2-based) embeddings

Based on our findings thus far, there appears to be a significant alignment between contextual embedding (i.e., induced by deep language models) and IFG’s brain embeddings which allowed us to predict better (above-nearest neighbor matching) newly-introduced words that were not included in the training. Next, we tested the ability of a symbolic-based (interpretable) model for zero-shot inference. To transform a symbolic model into a vector representation, we utilized^54^ to extract 75 symbolic (binary) features for every word within the text. These features include part of speech (POS) with 11 features, stop word, word shape with 16 features, types of prefixes with 19 dimensions, and types of suffixes with 28 dimensions. For a full list of all feature dimensions, see Supplementary Table 1. Next, we built a 75-dimensional (binary) vector for each word using these linguistic features. To match the dimension of the symbolic model and the embeddings model, we PCA the symbolic model to 50 dimensions. We obtained similar results with the 75d and 50d symbolic embeddings. We next ran the exact encoding analyses (i.e., zero-shot mapping) we ran using the contextual embeddings but using the symbolic model. The ability of the symbolic model to predict the activity for unseen words was greater than chance but significantly lower than contextual (GPT-2-based) embeddings (Fig. S7A). We did not find significant evidence that the symbolic embeddings generalize and better predict newly-introduced words that were not included in the training (above-nearest neighbor matching, red line in Fig. S7A). This means that the symbolic model can predict the activity of a word that was not included in the training data, such as the noun “monkey” based on how it responded to other nouns (like “table” and “car”) during training. However, we did not find evidence that it is able to interpolate and make new predictions about the representation (embedding) of a specific left-out noun (“monkey”) when used in the context of an unseen segment of the story, which we termed zero-shot inference. To enhance the symbolic model, we incorporated contextual information from the preceding three words into each vector, but adding symbolic context did not improve the fit (Fig. S7B). Lastly, the ability to predict above-nearest neighbor matching embedding using GPT-2 was found significantly higher of contextual embedding than symbolic embedding (Fig. S7C).

### Zero-shot decoding of individual words

In the preceding zero-shot encoding analysis, we successfully mapped the contextual embeddings into the brain embedding space of the IFG. Next, we reversed the mapping (i.e., decoding analysis) to map the brain embedding space into the contextual embedding space of GPT-2 (Fig. 1D). For this purpose, we used a two-step classification procedure^40^. Because this analysis requires multi-label classification (110 labels for each test fold), we trained a decoder using the same architecture introduced in our previous paper^40^. First, we trained a deep convolutional neural network to align the brain embedding of each word in the training folds to their corresponding contextual embedding (see Materials and Method and Supplementary Information). Next, we used the trained neural network to predict the contextual embeddings for the signal associated with each unseen word in the test fold. The cosine distance between the predicted and actual contextual embedding was used to classify the nearest word in the 110-word test set (i.e., zero-shot decoding). As with the encoding analysis, we repeated this procedure separately for different temporal shifts relative to work onset. We used the area under the receiver operating characteristic curve (ROC-AUC) to quantify the amount of information for each word. ROC-AUC of 0.5 indicates chance performance, and ROC-AUC of 1 indicates perfect classification among all test words.

**Fig. 3:**
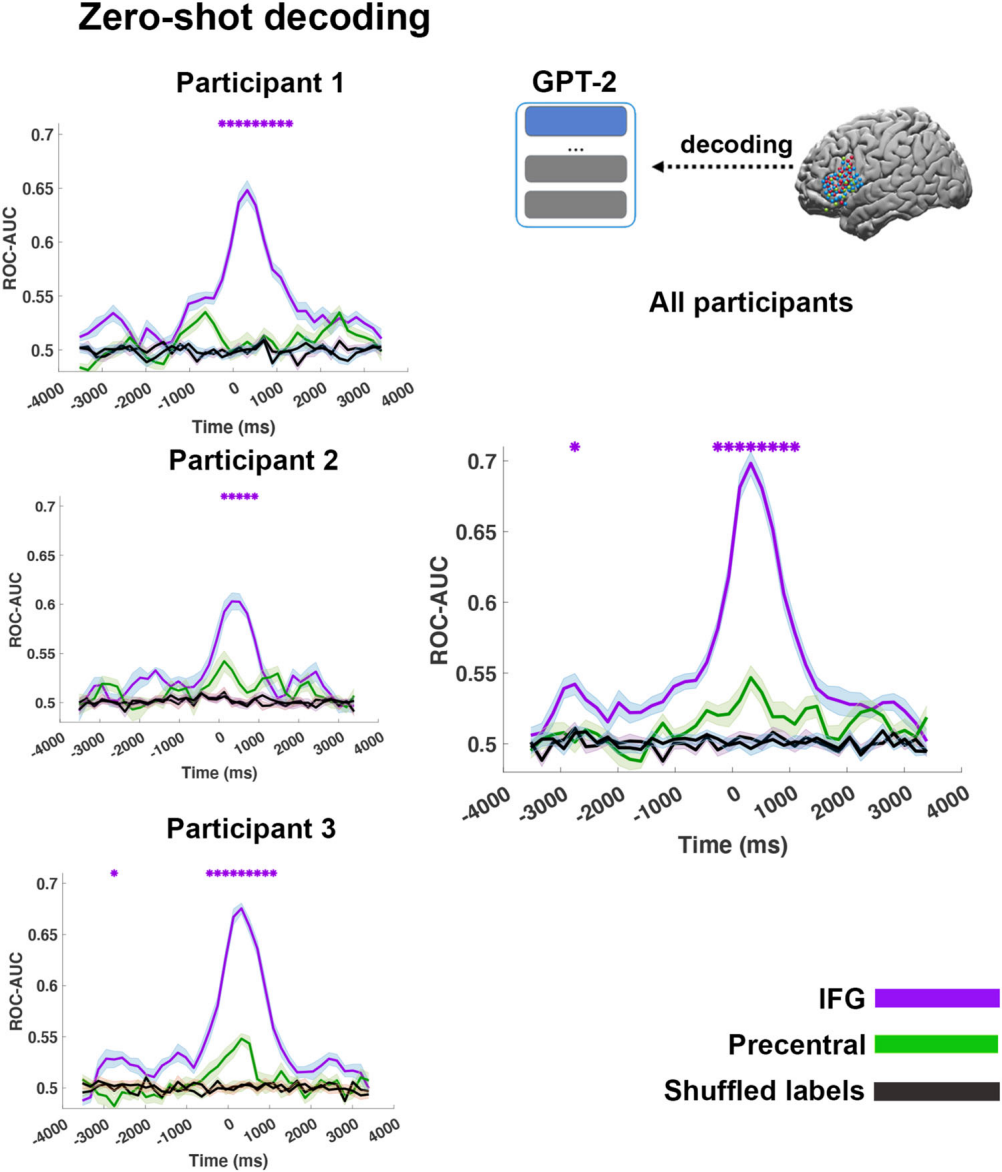
Zero-shot decoding of unseen words after aligning brain embeddings of the inferior frontal gyrus (IFG) and precentral gyrus to GPT-2 contextual embeddings. Average area under the receiver operating characteristic curve (ROC-AUC) for zero-shot word classification based on the predicted brain embeddings in IFG (purple line) and precentral gyrus (green line). All shaded lines represent standard error above and standard error below average. Zero-shot decoding was performed for each individual participant using brain embeddings. The classification is performed for all unseen words in each test fold, and performance is averaged across all ten test folds (the error bands indicate the standard error of the ROC-AUC scores across the folds). The classification was performed by computing the cosine distance between each predicted embedding and all other 110 words in the test fold. The black lines show the zero-shot classification between brain embeddings and shuffled contextual embeddings. In purple asterisks, we mark the significant difference, one-sided *p* value (*p* < 0.001), between the average ROC-AUC scores (*n* = 1100) based on the IFG and precentral embeddings, using paired sample permutation and Bonferroni correction for multiple comparisons.

Using zero-shot decoding, we could classify words well above-chance (Fig. 3). Decoding performance was significant at the group level, and we replicated the results in all three individuals. Peak classification was observed at a lag of roughly 320 ms after word onset with a ROC-AUC of 0.60, 0.65, and 0.67 in individual participants and 0.70 at the group level (Fig. 3, pink line). Shuffling the labels reduced the ROC-AUC to roughly 0.5 (chance level, Fig. 3 black lines). Running the same procedure on the precentral gyrus control area (Fig. 3, green line) yielded an AUC closer to the chance level (maximum AUC of 0.55). The marginal classification in the precentral gyrus may be attributed to the anatomical proximity to the IFG, functional properties peripherally related to language comprehension (e.g., articulation), or to the enhanced power of nonlinear models for aligning embedding spaces. We replicated these results on the set of fold-specific embedding (used for Fig. S7). We also ran the analysis for a linear model with a 200 ms window, equating to the encoding analysis, and replicated the results, albeit with a smaller effect (Fig. S8). Finally, to further substantiate the assertion of common geometric patterns with contextual embedding, we contrasted the performance of the decoding model based on contextual embedding (facilitated by GPT-2) with the performance of state-of-the-art non-contextual embedding (GloVe). The findings clearly demonstrated a substantial enhancement in performance when using contextual embedding (see Fig. S10).

## Discussion

How does the brain encode the contextual meaning of words in natural language? Our findings depart from the symbolic representations and rule-based syntactic operations of classical neurolinguistics. Using dense, high-resolution ECoG recordings, we sampled words’ continuous vector representation (brain embeddings) in a natural narrative within a well-localized language area, the IFG. Using zero-shot encoding and decoding, we demonstrate that IFG relies on continuous embeddings to represent words in natural contexts. Importantly, the brain embedding space shares common geometric properties with the contextual embedding space learned by DLMs. These common geometric patterns were sufficient to predict activity patterns in IFG for a new set of words not seen during training. This demonstrates that there is a mapping between the two spaces. Critically, the zero-shot predictions were precise enough to predict (interpolate) the embeddings of novel words more accurately than the most similar word (its nearest neighbor) in the training set. This means that the common geometric patterns allow for more precise interpolation than can be learned by the linear model by simply memorizing the training words. For example, it is possible to have an above-chance in performance in predicting the brain embedding associated with ‘monkey’ by basically predicting the brain embedding of a word with a similar meaning that appears in the training set (e.g., “baboon”). This would suggest that while there are some common geometric patterns, the mapping is not very precise. However, we showed that the predictions better match the actual test words than the nearest training words.

The zero-shot inference demonstrates that the electrode activity vectors predicted from the geometric embeddings closely correspond to the activity pattern for a given word in the electrode space. While most prior studies focused on the analyses of single electrodes, in this study, we densely sample the population activity, of each word, in IFG. These distributed activity patterns can be seen as points in high-dimensional space, where each dimension corresponds to an electrode, hence the term brain embedding. Similarly, the contextual embeddings we extract from GPT-2 for each word are numerical vectors representing points in high-dimensional space. Each dimension corresponds to one of 1600 features at a specific layer of GPT-2. GPT-2 effectively re-represents the language stimulus as a trajectory in this high-dimensional space, capturing rich syntactic and semantic information. The regression model used in the present encoding analyses estimates a linear mapping from this geometric representation of the stimulus to the electrode. However, it cannot nonlinearly alter word-by-word geometry, as it only reweights features without reshaping the embeddings’ geometry. Therefore, without common geometric patterns between contextual and brain embeddings in IFG, we could not predict (zero-shot inference) the brain embeddings for unseen left-out words not seen during training.

Zero-shot inference provides a principled way for testing the neural code for representing words in language areas. The zero-shot procedure removes information about word frequency from the model as it only sees a single instance of each word during training and evaluates model performance on entirely new words not seen during training. Therefore, the model must rely on the geometrical properties of the embedding space for predicting (interpolating) the neural responses for unseen words during the test phase. It is crucial to highlight the uniqueness of contextual embeddings, as their surrounding contexts rarely repeat themselves in dozens or even hundreds of words. Nonetheless, it is noteworthy that contextual embeddings for the same word in varying contexts exhibit a high degree of similarity^55^. Most vectors for contextual variations of the same word occupy a relatively narrow cone in the embedding space. Hence, splitting the unique words between the train and test datasets is imperative to ensure that the similarity of different contextual instances of the same word does not drive encoding and decoding performance. This approach ensures that the encoding and decoding performance does not result from a mere combination of memorization acquired during training and the similarity between embeddings of the same words in different contexts.

Our results indicate that contextual embedding space better aligns with the neural representation of words in the IFG than the static embedding space used in prior studies^22–24^. A previous study suggested that static word embeddings can be conceived as the average embeddings for a word across all contexts^40,56^. Thus, a static word embedding space is expected to preserve some, but not all, of the relationships among words in natural language. This can explain why we found significant yet weaker interpolation for static embeddings relative to contextual embeddings. Furthermore, the reduced power may explain why static embeddings did not pass our stringent nearest neighbor control analysis. Together, these results suggest that the brain embedding space within the IFG is inherently contextual^40,56^. While the embeddings derived from the brain and GPT-2 have similar geometry, they are certainly not identical. Testing additional embedding spaces using the zero-shot method in future work will be needed to explore further the neural code for representing language in IFG.

We are not suggesting that classical psycholinguistic grammatical notions should be disregarded. In this paper, we define symbolic models as interpretable models that blend symbolic elements (such as nouns, verbs, adjectives, adverbs, etc.) with hard-coded rule-based operations. On the other hand, deep language models are statistical models that learn language from real-world data, often without explicit prior knowledge about language structure. If symbolic terms encapsulate some aspects of linguistic structure, we anticipate statistical learning-based models will likewise embed these structures^31,32^. Indeed^8,57–60^, succeeded in extracting linguistic information from contextual embeddings. However, it is important to note that although large language models may capture soft rule-like statistical regularities, this does not transform them into rule-based symbolic systems. Deep language models rely on statistical rather than symbolic foundations for linguistic representations. By analyzing language statistics, these models embed language structure into a continuous space. This allows the geometry of the embedded space to represent the statistical structure of natural language, including its regularities and peculiar irregularities.

We did not find statistically significant evidence for symbolic-based models performing zero-shot inference and delivering better predictions (above-nearest neighbor matching), for newly-introduced words that were not included in the training. However, the ability to predict above-nearest neighbor matching embedding using GPT-2 was found significantly higher in contextual embedding than in symbolic embedding. This suggests that deep language-model-induced representations of linguistic information are more aligned with brain embeddings sampled from IFG than symbolic representation. This discovery alone is not enough to settle the argument, as there may be new symbolic-based models developed in future research to enhance zero-shot inference while still utilizing a symbolic language representation.

While we found evidence for common geometric patterns between brain embeddings derived from IFG and contextual embedding derived from GPT-2, our analyses do not assess the dimensionality of the embedding spaces^61^. In this work, we reduce the dimensionality of the contextual embeddings from 1600 to 50 dimensions. We demonstrate a common continuous-vectorial geometry between both embedding spaces in this lower dimension. To assess the latent dimensionality of the brain embeddings in IFG, we need a denser sampling of the underlying neural activity and the semantic space of natural language^61^.

Why are there common geometric patterns of language in DLMs and the human brain? After all, there are fundamental differences between the way DLMs and the human brain learn a language. For example, DLMs are trained on massive text corpora containing millions or even billions of words. The sheer volume of data used to train these models is equivalent to what a human would be exposed to in thousands of years of reading and learning. Furthermore, current DLMs rely on the transformer architecture, which is not biologically plausible^62^. Deep language models should be viewed as statistical learning models that learn language structure by conditioning the contextual embeddings on how humans use words in natural contexts. If humans, like DLMs, learn the structure of language from processing speech acts, then the two representational spaces should converge^32,61^. Indeed, recent work has begun to show how implicit knowledge about syntactic and compositional properties of language is embedded in the contextual representations of deep language models^9,63^. The common representational space suggests that the human brain, like DLMs, relies on overparameterized optimization to learn the statistical structure of language from other speakers in the natural world^32^.

We acknowledge that the results were obtained from three patients with dense recordings of their IFG. The dense grid research technology is only employed by a few groups worldwide, especially chronically, we believe that in the future, more of this type of data will be available. The results should be replicated using information collected from larger samples of participants with dense recordings.

To conclude, the alignment between brain embeddings and DLM contextual embeddings, combined with accumulated evidence across recent papers^35,37,38,40,61^ suggests that the brain may rely on contextual embeddings to represent natural language. The move from a symbolic representation of language to a continuous contextual embedding representation is a conceptual shift for understanding the neural basis of language processing in the human brain.

## Methods

### Ethical oversight

Princeton University and New York University School of Medicine’s respective Institutional Review Boards approved the Studies.

### Participants

Three patients (two females (gender assigned based on medical record); 24–48 years old) with treatment-resistant epilepsy undergoing intracranial monitoring with subdural grid and strip electrodes for clinical purposes participated in the study. No statistical method was used to predetermine the sample size. Three study participants consented to have an FDA-approved hybrid clinical-research grid implanted that includes additional electrodes in between the standard clinical contacts. The hybrid grid provides a higher spatial coverage without changing clinical acquisition or grid placement. Each participant provided informed consent following protocols approved by the New York University Grossman School of Medicine Institutional Review Board. Patients were informed that participation in the study was unrelated to their clinical care and that they could withdraw from the study without affecting their medical treatment.

### Stimulus

Participants were presented with a 30-min auditory story stimulus, “So a Monkey and a Horse Walk Into a Bar: Act One, Monkey in the Middle” taken from the *This American Life* podcast. The onset of each word was marked using the Penn Phonetics Lab Forced Aligner^64^ and manually validated and adjusted (if necessary). Data acquisition and preprocessing.

### Preprocess

First, large spikes exceeding four quartiles above and below the median were removed, and replacement samples were imputed using cubic interpolation. Second, the data were re-referenced using common average referencing. Third, six-cycle wavelet decomposition was used to compute the high-frequency broadband (HFBB) power in the 70–200 Hz band, excluding 60, 120, and 180 Hz line noise. In addition, the HFBB time series of each electrode was log-transformed and z-scored. Fourth, the signal was smoothed using a Hamming window with a kernel size of 50 ms. The filter was applied in both the forward and reverse directions to maintain the temporal structure. Additional preprocessing details can be found in prior work^40^.

Data were preprocessed using Matlab 2019b and The Fieldtrip toolbox. Data was analyzed using Python packages are specified here https://github.com/hassonlab/247-main/blob/main/env.yml. Brain plots were done using a toolbox for MATLAB available at (https://github.com/HughWXY/ntools_elec). All scripts for analyses are available at: All the scripts for analyses can be found at: https://github.com/orgs/hassonlab/repositories.

### Contextual embedding

We extracted contextualized word embeddings from GPT-2 using the Hugging Face environment^65^. We first converted the words from the raw transcript (including punctuation and capitalization) to tokens comprising whole words or sub-words (e.g., there’s → there’s). We used a sliding window of 1024 tokens, moving one token at a time, to extract the embedding for the final word in the sequence (i.e., the word and its history). We extracted the activity of the final hidden layer of GPT-2 (which has 48 hidden layers). The contextual embedding of a word is the activity of the last hidden layer given all the words up to and not including the word of interest (in GPT-2, the word is predicted using the last hidden state). The original dimensionality of the embedding is 1600, and it is reduced to 50 using PCA.

### Brain embedding

We extracted brain embeddings for specific ROIs by averaging the neural activity in a 200 ms window for each electrode in the ROI. This means that if there are *N* electrodes in a specific ROI for a specific patient, then, for each lag (ranging from −4 s to +4 s in 25 ms shifts relative to word onset), there will be an *N*-dimensional embedding, where each feature is the averaged neural activity of a specific electrode of the neural recordings in a window of 200 ms (102-time points) centered at the lag.

### Zero-shot encoding model

Linear encoding models were estimated at each lag relative to word onset to predict the brain embedding for each word from the corresponding contextual embedding. Before fitting the encoding model, we smoothed the signal using a rolling 200-ms window (i.e., averaged every consecutive 102 samples). We used a tenfold cross-validation procedure, ensuring that for each cross-validation fold, the model was estimated from a subset of unique training words and evaluated on a nonoverlapping subset of unique, held-out test words: the words and their corresponding brain embeddings were split into a training set (90% of the unique words) for model estimation and a test set (10% of the unique words) for model evaluation (zero-shot analysis). Only one instance of each word was included in the analysis. We used ordinary least squares (OLS) multiple linear regression for each cross-validation fold to estimate a weight vector (for the 50-dimensional model feature space) based on the training words. We then used those weights to predict the neural responses at each electrode (comprising a brain embedding across electrodes) for the test words. We evaluated model performance by computing the correlation between the predicted brain embedding and the actual brain embedding (i.e., the distributed neural activity pattern) for each held-out test word; we then averaged these correlations across test words. This procedure was repeated in full at 321 lags at 25-ms increments from −4000 ms to 4000 ms relative to word onset. As a control analysis, for each test word, we used the nearest contextual embedding (in terms of cosine distance) from the training set to predict the brain embedding for the test word.

### Zero-shot decoding model

We used a decoding model to classify unseen words from the corresponding brain embeddings. The neural signals were first averaged per electrode in ten 62.5-ms bins spanning 625 ms for each lag. Each bin had 32 data points (the neural recording sampling rate was 512 Hz). We used a tenfold cross-validation procedure, ensuring that for each cross-validation fold, the decoding model was trained on a subset of unique training words and evaluated on a nonoverlapping subset of unique, held-out test words: the words and their corresponding brain embeddings were split into a training set (90% of the unique words) for model estimation and a test set (10% of the unique words) for model evaluation (zero-shot analysis). A neural network decoder (see architecture in Supplementary Information) was trained to predict the contextual embedding for each word from the corresponding brain embedding at a specific lag. Eight training folds were used for training the decoder (training set), one fold was used for early stopping (development set), and one fold was used to assess model generalization (test set). When predicting the embedding, the neural network was optimized to minimize the mean squared error (MSE).

First, we computed the cosine similarity between the predicted contextual embedding and all the unique contextual embeddings in the dataset (Fig. 3 blue lines). We used a softmax transformation on these scores (logits). For each label, we used these logits to evaluate whether the decoder predicted the matching word and computed an ROC-AUC for the label. Each test word is evaluated against the other test words in that particular test set in this evaluation strategy. To improve the decoder’s performance, we implemented an ensemble of models. We independently trained six classifiers with randomized weight initializations and randomized the batch order supplied to the neural network for each lag. This procedure generated six predicted embeddings. Thus, we repeated the distance calculation from each word label six times for each predicted embedding. These six values were averaged and used to compute the ROC-AUC.

### Statistical significance

We used a bootstrap hypothesis test to assess the statistical significance of the correlations between predicted and actual brain embeddings. The test statistic reported for each lag is the average of the correlations between the predicted brain embedding and actual brain embedding across all test words. We then resampled these correlations across words with replacement (5000 bootstrap samples) to generate a bootstrap distribution around the mean correlation. We then computed a *p* value based on the null hypothesis that the correlation is zero. This procedure was repeated for each lag (321 lags), and we controlled the false discovery rate (FDR) at *q* = 0.01^66^.

To test whether there was a significant difference between the performance of the model using the actual contextual embedding for the test words compared to the performance using the nearest word from the training fold, we performed a permutation test. At each iteration, we permuted the differences in performance across words and assigned the mean difference to a null distribution. We then computed a *p* value for the difference between the test embedding and the nearest training embedding based on this null distribution. This procedure was repeated to produce a *p* value for each lag and we corrected for multiple tests using FDR.

To compare the difference between classifier performance using IFG embedding or precentral embedding for each lag, we used a paired sample *t*-test. We compared the AUC of each word classified with the IFG or precentral embedding for each lag. We used Bonferroni correction to account for the multiple comparisons.

### Reporting summary

Further information on research design is available in the Nature Portfolio Reporting Summary linked to this article.

## Supplementary information

Supplementary Information

Reporting Summary

## Data Availability

The neural data are available under restricted access, for it may contain sensitive information. Access can be obtained upon request from Ariel Goldstein (the corresponding author). The data underlying the figures can be found at https://zenodo.org/records/10658831^67^.

## Code availability

We provide the code for replicating the core analyses of this manuscript: https://github.com/hassonlab/247-plotting/blob/main/scripts/tfspaper_zeroshot.ipynb^68^.
