# Author Correction: Alignment of brain embeddings and artificial contextual embeddings in natural language points to common geometric patterns

**DOI:** 10.1038/s41467-024-52626-6

**Published:** 2024-10-01

**Authors:** Ariel Goldstein, Avigail Grinstein-Dabush, Mariano Schain, Haocheng Wang, Zhuoqiao Hong, Bobbi Aubrey, Samuel A. Nastase, Zaid Zada, Eric Ham, Amir Feder, Harshvardhan Gazula, Eliav Buchnik, Werner Doyle, Sasha Devore, Patricia Dugan, Roi Reichart, Daniel Friedman, Michael Brenner, Avinatan Hassidim, Orrin Devinsky, Adeen Flinker, Uri Hasson

**Affiliations:** 1 Business School, Data Science department and Cognitive Department, Hebrew University, Jerusalem, Israel; 2 Google Research, Tel Aviv, Israel; 3 Department of Psychology and the Neuroscience Institute, Princeton University, Princeton, NJ USA; 4 New York University Grossman School of Medicine, New York, NY USA; 5 Faculty of Industrial Engineering and Management, Technion, Israel Institute of Technology, Haifa, Israel; 6 School of Engineering and Applied Science, Harvard University, Cambridge, MA USA; 7 New York University Tandon School of Engineering, Brooklyn, NY USA

**Keywords:** Neural encoding, Neural decoding, Language

**Correction to:** *Nature Communications* 10.1038/s41467-024-46631-y, published online 30 March 2024

The original version of this Article had an error in the author list, with Mariano Schain appearing twice.

The original author list was: Ariel Goldstein, Avigail Grinstein-Dabush, Mariano Schain, Haocheng Wang, Zhuoqiao Hong, Bobbi Aubrey, Mariano Schain, Samuel A. Nastase, Zaid Zada, Eric Ham, Amir Feder, Harshvardhan Gazula, Eliav Buchnik, Werner Doyle, Sasha Devore, Patricia Dugan, Roi Reichart, Daniel Friedman, Michael Brenner, Avinatan Hassidim, Orrin Devinsky, Adeen Flinker & Uri Hasson

The corrected author list is: Ariel Goldstein, Avigail Grinstein-Dabush, Mariano Schain, Haocheng Wang, Zhuoqiao Hong, Bobbi Aubrey, Samuel A. Nastase, Zaid Zada, Eric Ham, Amir Feder, Harshvardhan Gazula, Eliav Buchnik, Werner Doyle, Sasha Devore, Patricia Dugan, Roi Reichart, Daniel Friedman, Michael Brenner, Avinatan Hassidim, Orrin Devinsky, Adeen Flinker & Uri Hasson

This has been corrected in both the PDF and HTML versions of the Article.

