# Supplementary material for: Alignment of brain embeddings and artificial contextual embeddings in natural language points to common geometric patterns
Source: Nat Commun. 2024 Mar 30;15:2768. doi: 10.1038/s41467-024-46631-y (PMC10980748; doi:10.1038/s41467-024-46631-y)
Supplement: Supplementary file 1 — Supplementary Information [file 41467_2024_46631_MOESM1_ESM.pdf]

Alignment of Brain Embeddings and Artificial Contextual Embeddings in Natural Language Points to Common Geometric Patterns

Authors: Ariel Goldstein[1,2*], Avigail Dabush[2], Haocheng Wang[3], Zhuoqiao Hong[3], Bobbi Aubrey[3,4], Mariano Schain[2], Samuel A. Nastase[3], Zaid Zada[3], Eric Ham[3], Amir Feder[2], Harshvardhan Gazula[3], Eliav Buchnik[2], Werner Doyle[4], Sasha Devore[4], Patricia Dugan[4], Roi Reichart[5] ,Daniel Friedman[4], Michael Brenner[2,6], Avinatan Hassidim[2], Orrin Devinsky[4], Adeen Flinker[4,7], Uri Hasson[2,3]

Affiliations:
[1] Business School, Data Science department and Cognitive Department, Hebrew University, Jerusalem, Israel

[2] Google Research, Tel Aviv, Israel

[3] Department of Psychology and the Neuroscience Institute, Princeton University, Princeton, NJ, USA

[4] New York University Grossman School of Medicine, New York, NY, USA

[5] Faculty of Industrial Engineering and Management, Technion, Israel Institute of Technology, Haifa, Israel

[6] School of Engineering and Applied Science, Harvard University, Cambridge, MA , USA

[7] New York University Tandon School of Engineering, Brooklyn, NY, USA

* Corresponding author Ariel Goldstein. Email: ariel.y.goldstein@mail.huji.ac.il

Precentral

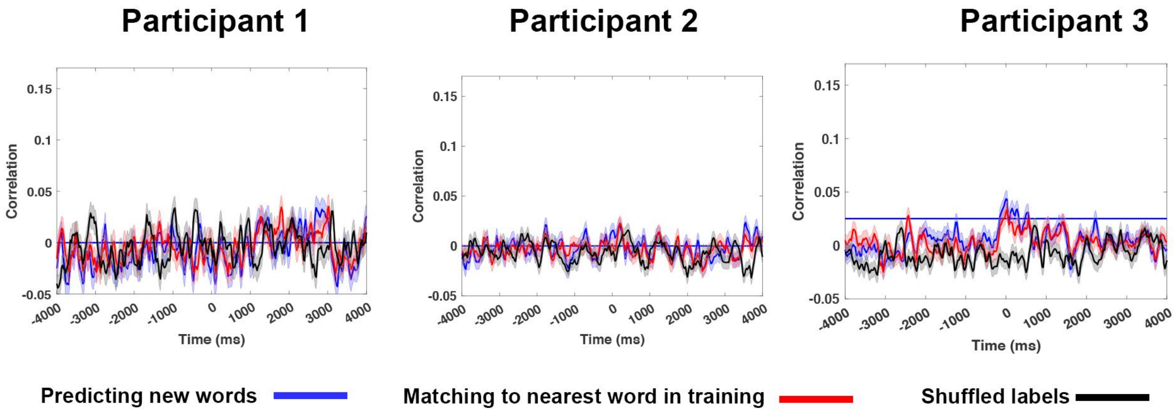

Figure S1. Zero-shot encoding between the contextual and brain embeddings of the precentral gyrus (control area) for each patient. Zero-shot encoding is evaluated at each lag (-4000 to 4000 ms). All shaded lines represent standard error above and standard error below average. There was no statistically significant difference between the encoding between near neighbor and the actual word.

Precentral and postcentral

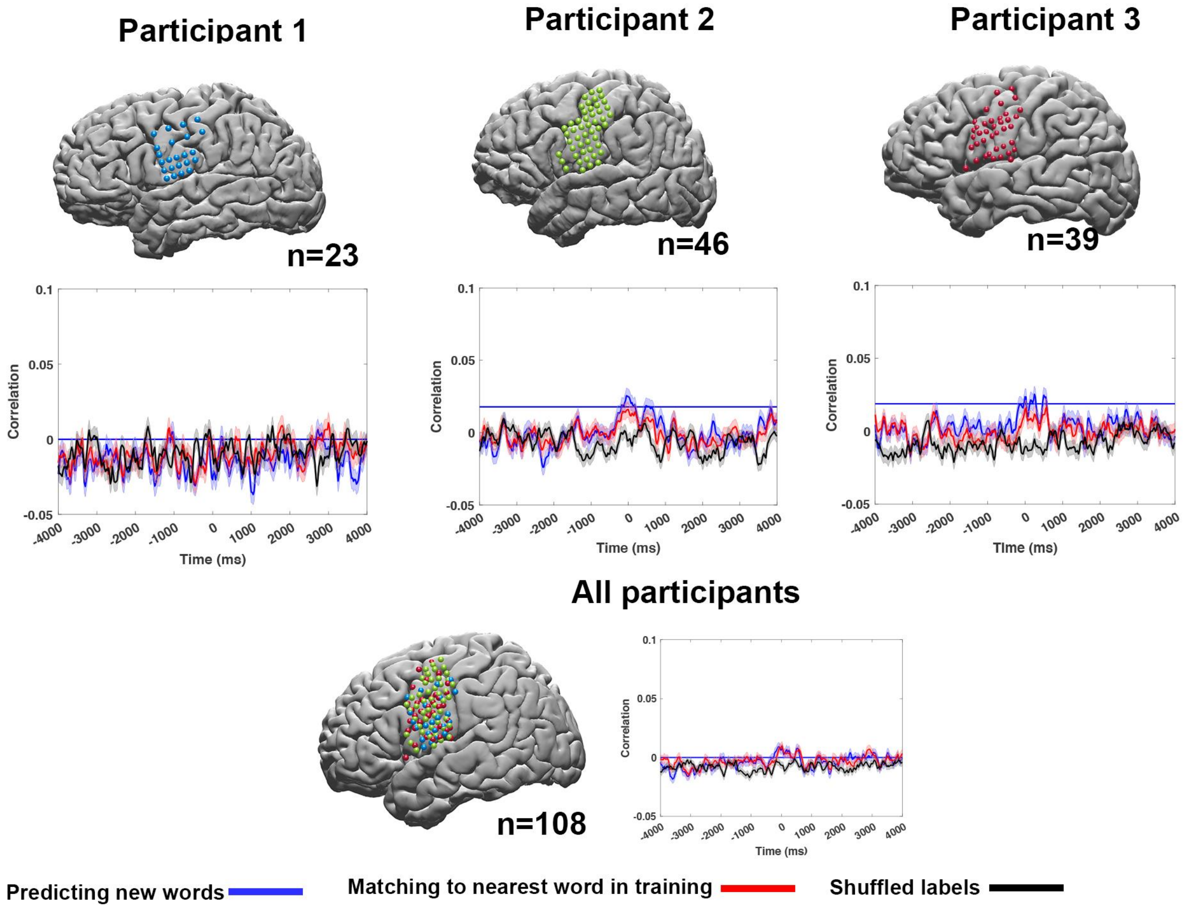

Figure S2. In order to control for the increased number of electrodes in the IFG we collapsed the precentral and postcentral gyrus and re-ran the encoding analysis. All shaded lines represent standard error above and standard error below average. There was no statistically significant difference between the original test set and the nearest neighbor from the training set.

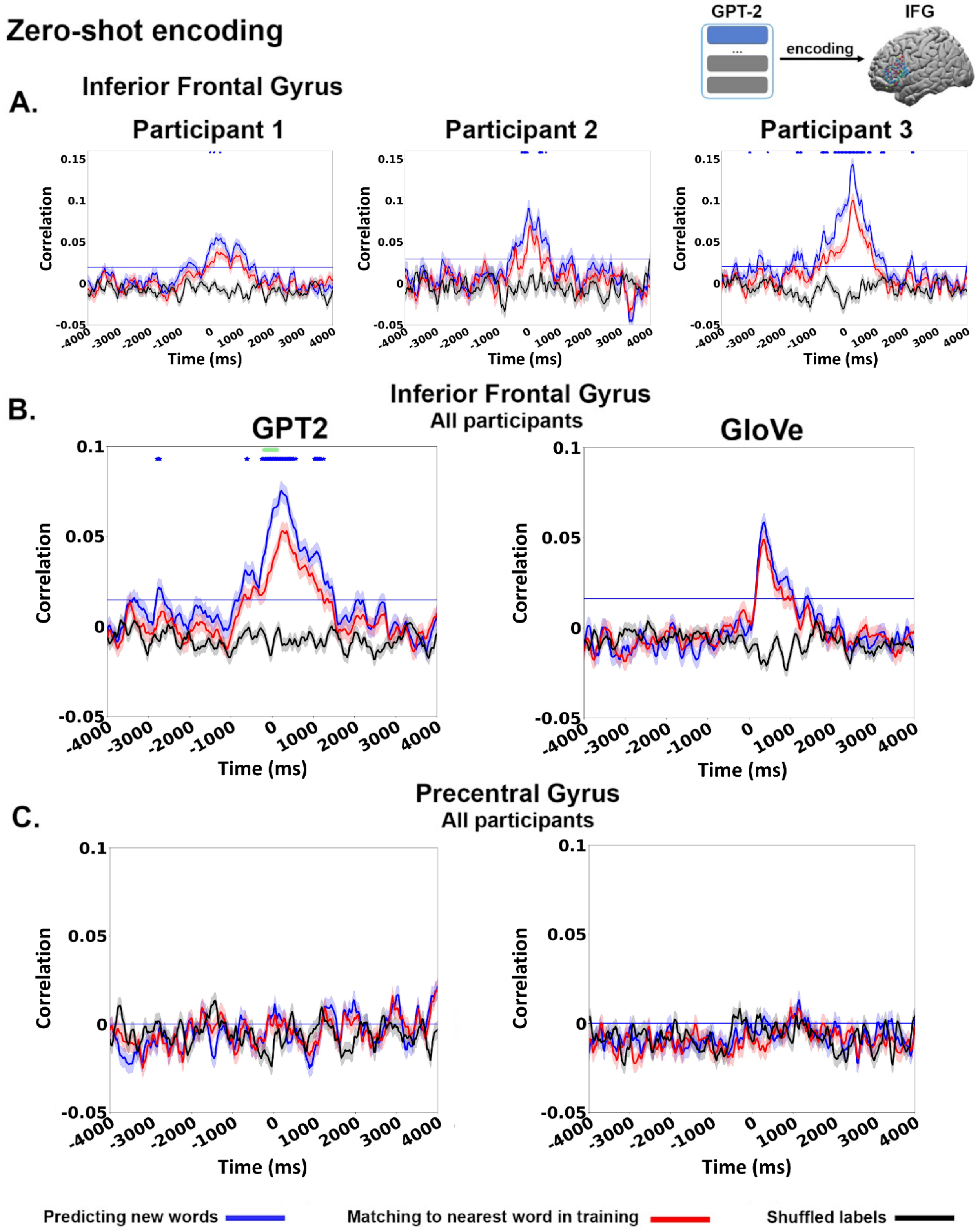
Zero-shot encoding
GPT-2
encoding
IFG
A.
Inferior Frontal Gyrus
Participant 1
Participant 2
Participant 3
Correlation
0.15
0.1
0.05
0
-0.05
-4000 -3000 -2000 -1000 0 1000 2000 3000 4000
Time (ms)
B.
Inferior Frontal Gyrus
All participants
GPT2
GloVe
C.
Precentral Gyrus
All participants
Predicting new words
Matching to nearest word in training
Shuffled labels

Figure S3. Zero-shot encoding using fold-specific contextual embeddings. We replicate the zero-shot encoding results using a more strict cross-validation scheme where contextual embeddings are extracted separately for each cross-validation fold (n=1100). This analysis controls for any possible leakage of contextual information across folds and reinforces the claim that GPT-2 geometry allows for the generalization of truly unseen words. All shaded lines represent standard error above and standard error below average. The blue asterisks represent a significant difference (one-sided, FDR corrected, q<0.01) between the correlation with the actual contextual embeddings (blue line) and the correlation with the nearest embedding from the training set (red line).

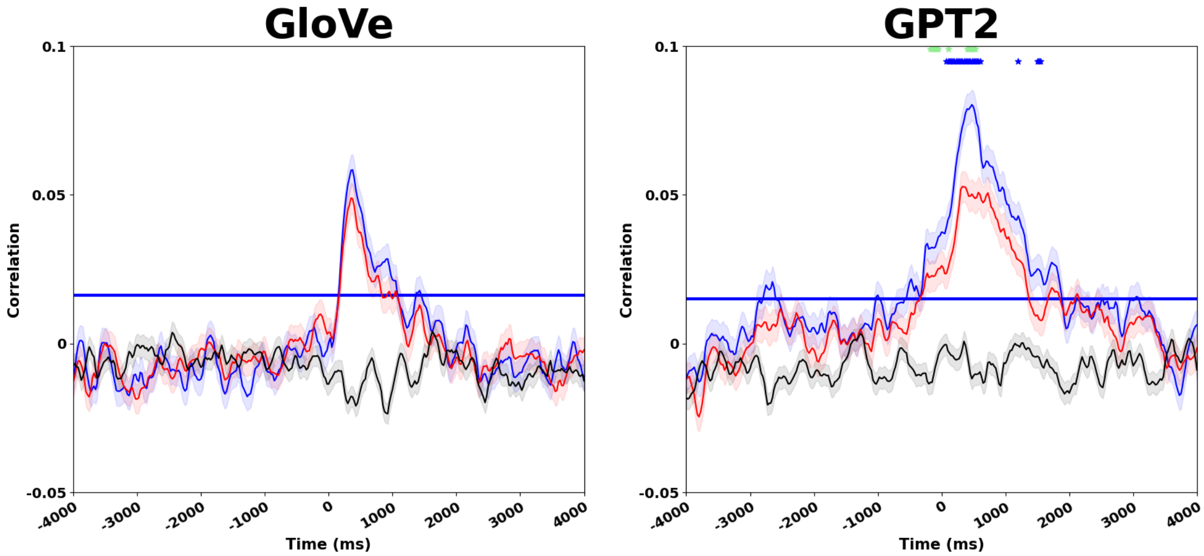

Figure S4. Reproducing the results, for contextual embedding that include the target word. Indicating common geometric patterns between brain embedding and contextual embedding induced by GPT-2 (blue asterisks); and higher alignment between brain embeddings and contextual embedding than brain embedding and GloVe embeddings (green asterisks). All shaded lines represent standard error above and standard error below average. The blue asterisks represent a significant difference (n=1100, one-sided, FDR corrected, q<0.01) between the correlation with the actual contextual embeddings (blue line) and the correlation with the nearest embedding from the training set (red line).

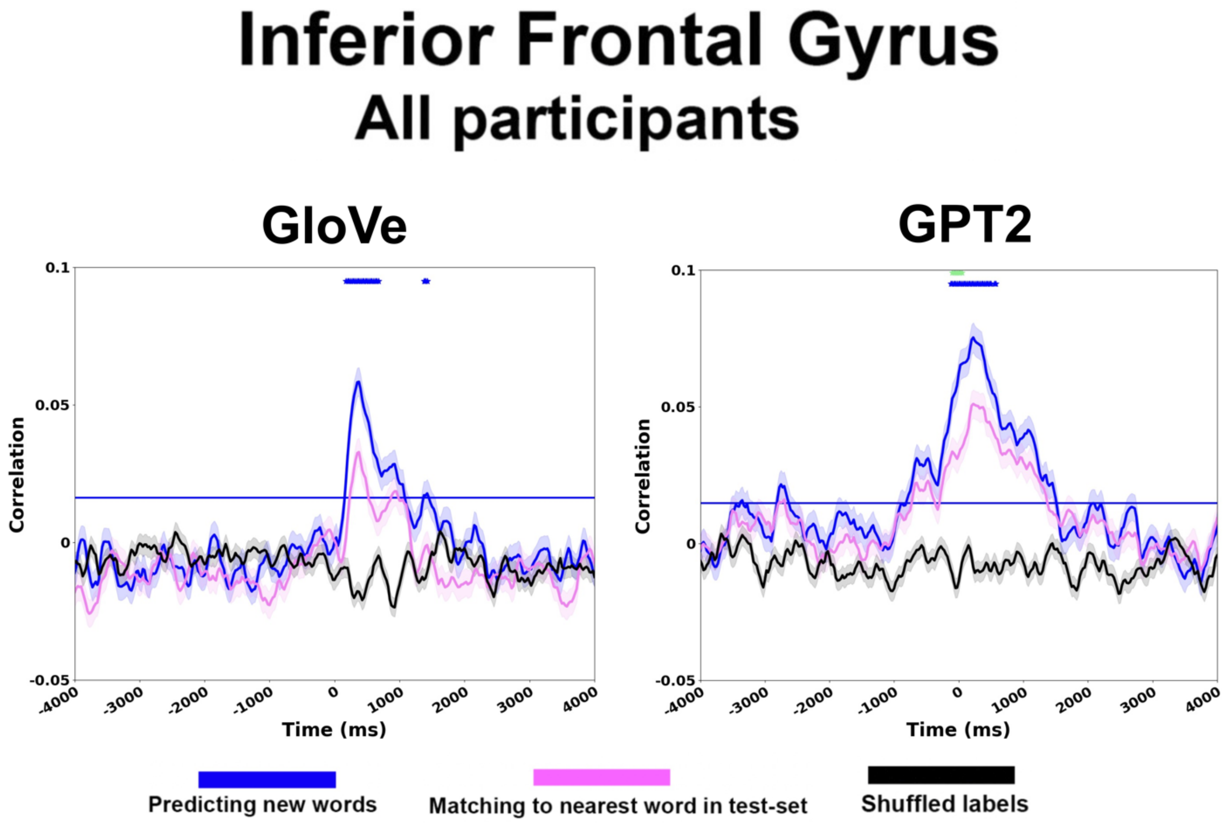

Figure S5. GPT-2 and GloVe predictions better match the target word than the nearest word from the test set, and GPT-2 better predicts than GloVe (Inspired by Mitchell and colleagues). We re-ran our analysis using static, non-contextual GloVe vectors. However, instead of using the nearest word from the training set as control, we used the nearest word from the test set. This follows the logic of Mitchell and colleagues, as they also used only unseen words in their analysis. Indeed, for this case, we do get a significant difference (blue asterisks; $p<0.01$; FDR corrected) in the GloVe conditions. However, we get an even higher difference for the GPT-2 condition (blue asterisks;$p<0.01$; FDR corrected); the within-comparison of the differences suggests GPT-2 has a bigger difference between the target word embedding and test-set-nearest word embedding than the difference of GloVe target word and GloVe test-set-nearest word embedding (green asterisks;$p<0.01$; FDR corrected; $n=1100$). All shaded lines represent standard error above and standard error below average.

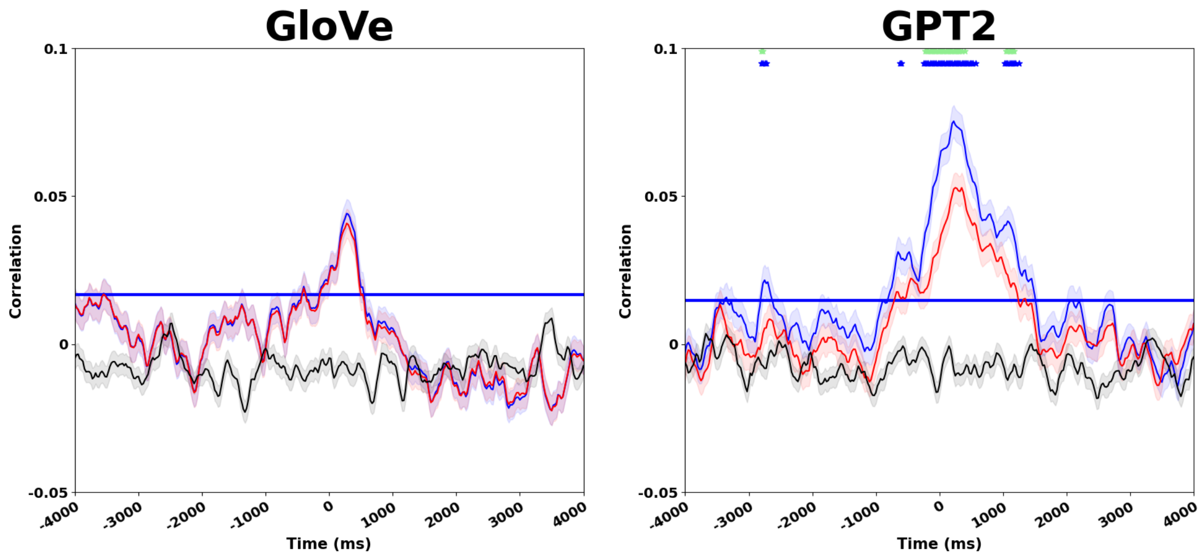

Figure S6. Concatenating previous words does not reproduce the effect of contextual embeddings. We concatenated GloVe embeddings for the 10 words that precede the target word to construct pseudo-contextual embeddings and reproduced the encoding analysis (left plot). GPT-2 contextual embeddings have a higher correlation with the brain embeddings (blue line, blue asterisk; $p<0.01$; FDR corrected), and the difference between the target embedding and the nearest neighbor from the training set (red line) is higher for GPT-2 than GloVe (green asterisks; $p<0.01$; FDR corrected; n=1100). All shaded lines represent standard error above and standard error below average.

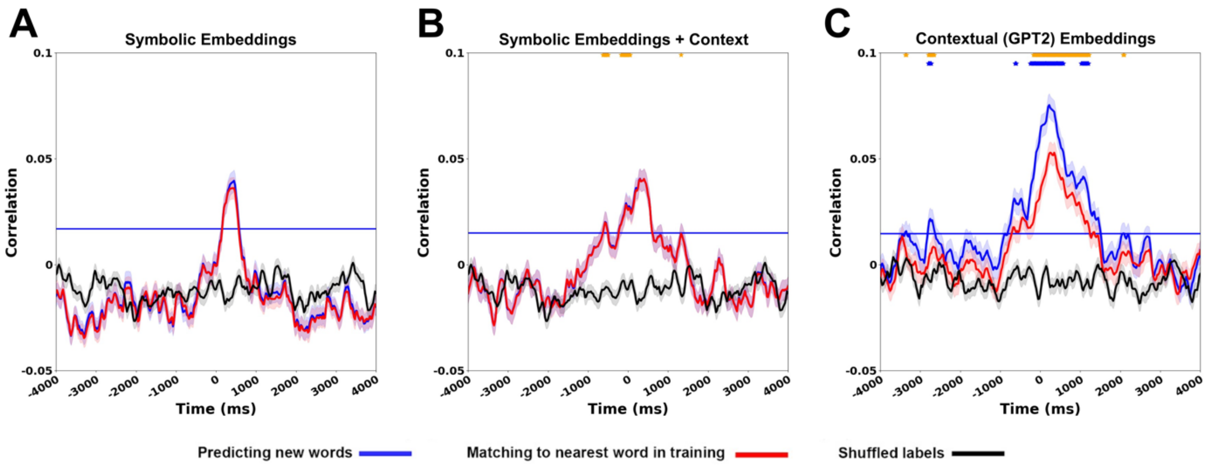

Figure S7. Symbolic embeddings do not reproduce the effect of contextual (GPT2-based) embeddings. (**A**) Zero-shot encoding between the symbolic and brain embeddings in IFG across all four patients. The solid blue line shows the average correlation between the predicted and actual brain embeddings in IFG for all words across all test sets. The red line shows the zero-shot encoding with the symbolic embedding for the word from the training set that is most similar (nearest neighbor) to each test word. The black line shows the zero-shot encoding between shuffled symbolic embeddings and the brain embeddings. (**B**) To enhance the symbolic model, we incorporated contextual information from the preceding three words into each vector. To that end, we concatenated the symbolic embeddings for the target word with the three symbolic embeddings of the preceding three words. Next, we replicated the zero-shot encoding analysis. Orange asterisks represent a significant difference between symbolic based correlation (in panel A) and contextual-symbolic based correlation. (**C**) For comparison, we present the same zero-shot encoding with contextual embedding derived from GPT2, as in Figure 2B. The ability to predict above-nearest neighbor matching embedding using GPT-2 was found significantly higher in contextual embedding than symbolic embedding (B). Orange asterisks represent a significant difference between GPT-2 based correlation and contextual-symbolic based (in panel B) correlation (one sided permutation test, n=1100, FDR corrected; $q<0.01$). All shaded lines represent standard error above and standard error below average.

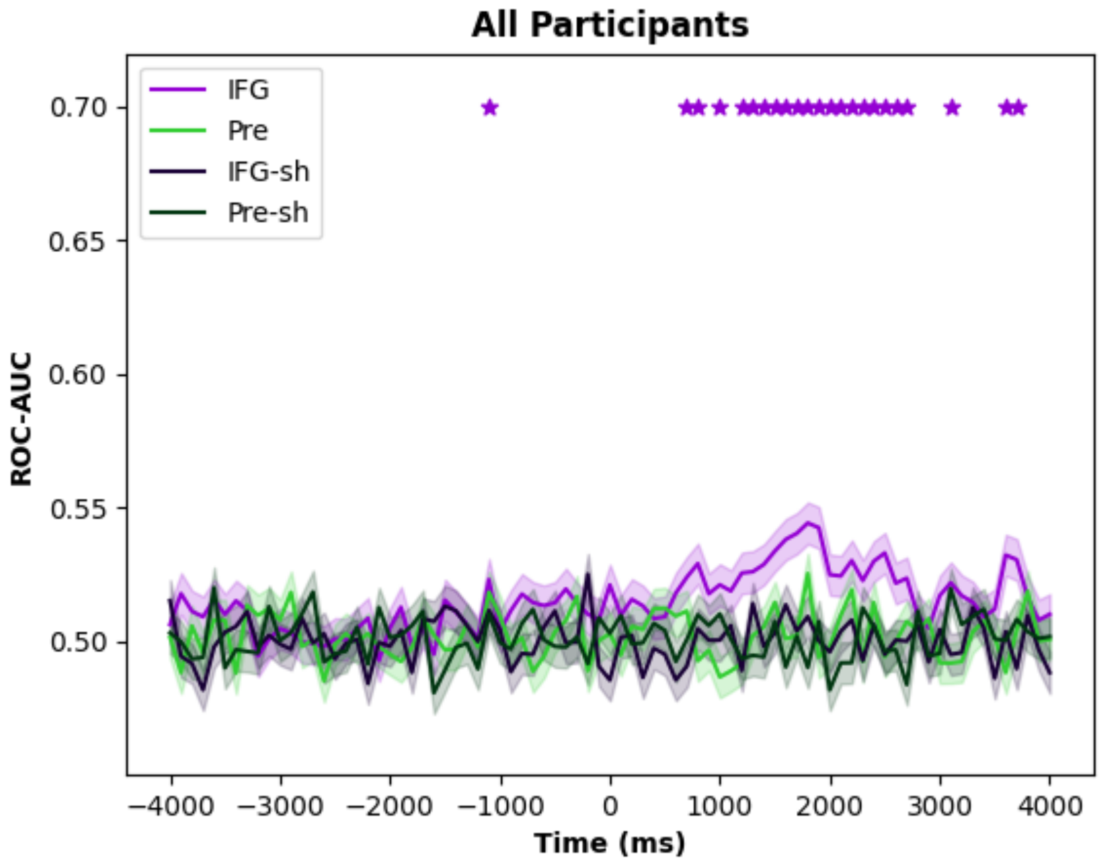

Figure S8. Replicating the results using contextual embeddings that are fold-specific. With similar attributes as the encoding analysis: Linear model, 200 ms input window, and infold embedding. All shaded lines represent standard error above and standard error below average. In purple asterisks, we mark the significant difference, one-sided p-value ($p<0.001$), between the average ROC-AUC scores ($n=1100$) based on the IFG and precentral embeddings, using paired sample permutation and Bonferroni correction for multiple comparisons.

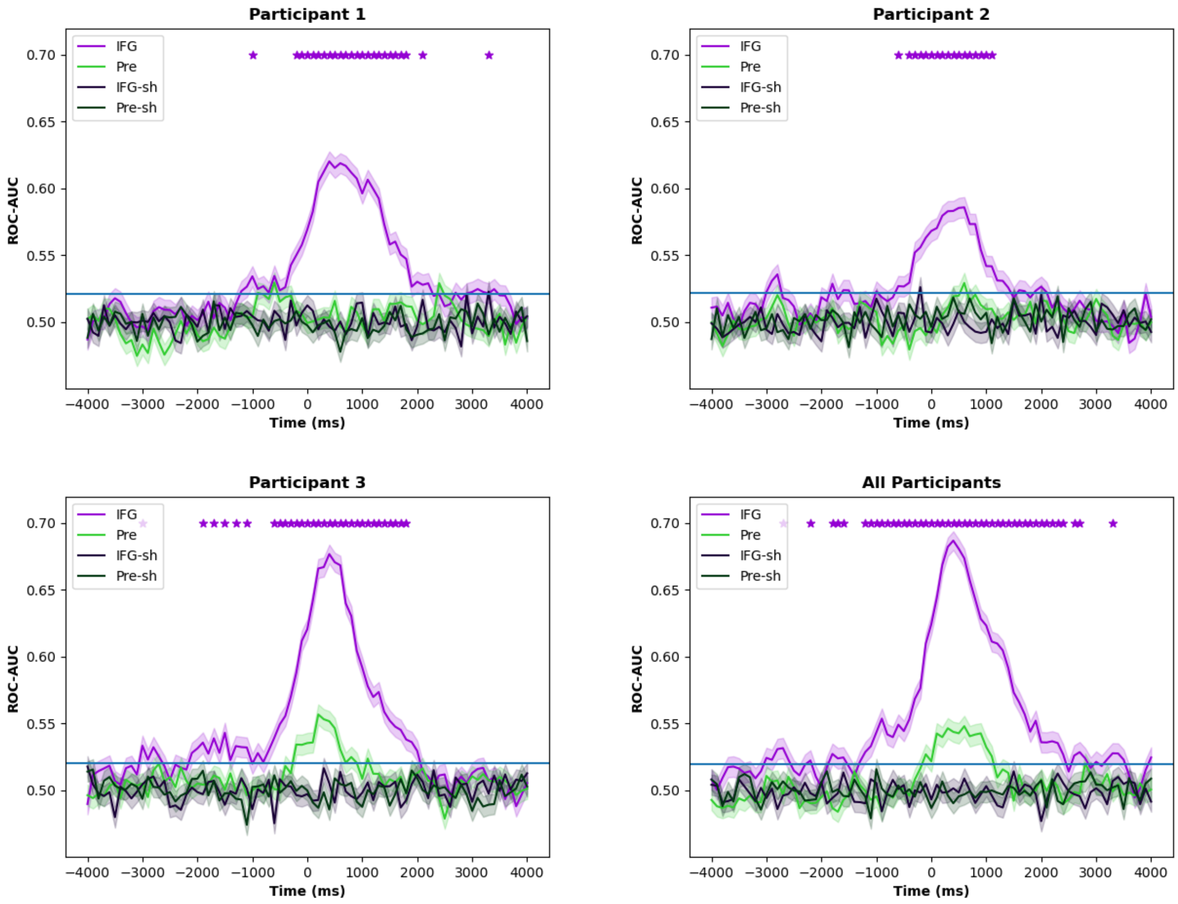

Figure S9. Replicating the results using contextual embeddings that are fold-specific. Controlling for the possible alternative explanation that the results obtained in Fig. 3 are due to a leak between folds during embedding extraction. All shaded lines represent standard error above and standard error below average. In purple asterisks, we mark the significant difference, one-sided p-value ($p<0.001$), between the average ROC-AUC scores ($n=1100$) based on the IFG and precentral embeddings, using paired sample permutation and Bonferroni correction for multiple comparisons.

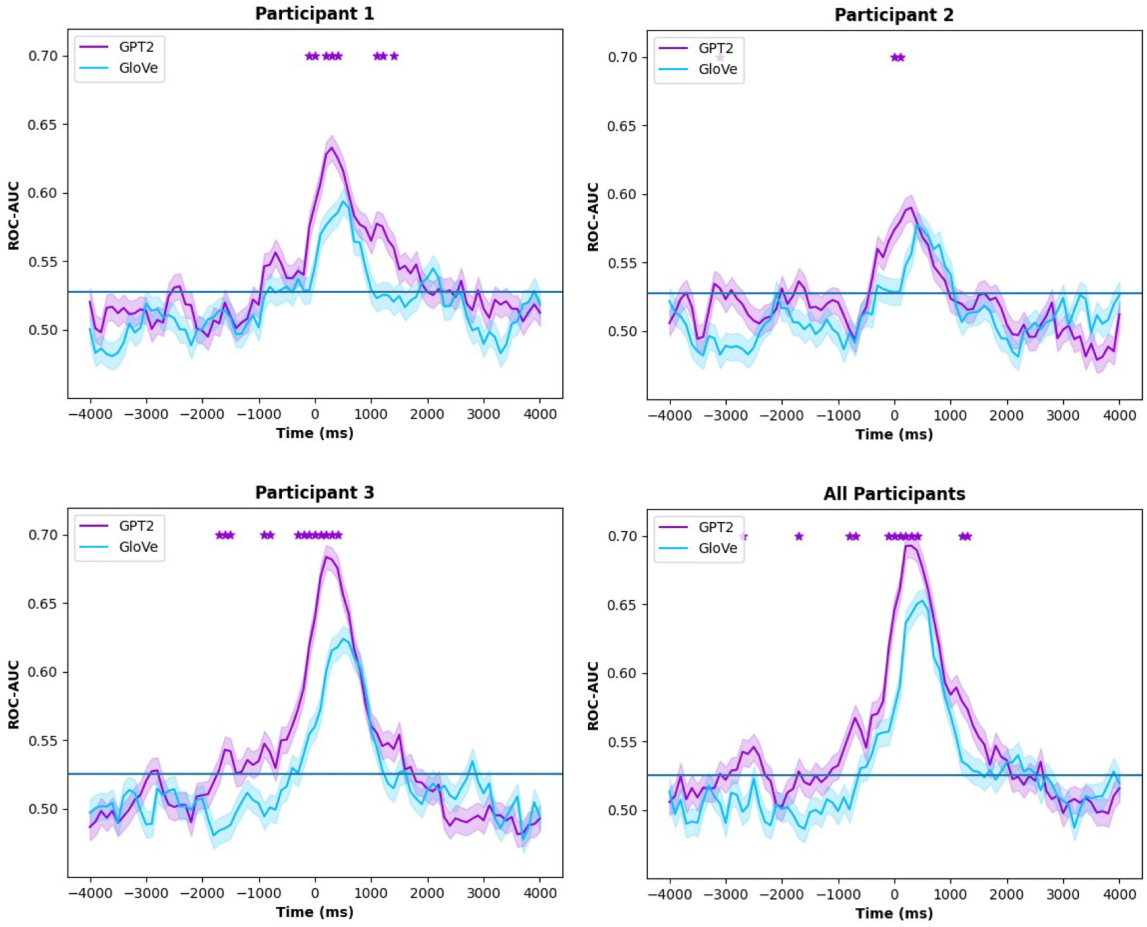

Figure S10. We compared the decoding performance of zero-shot classification by utilizing both static (non-contextual) embedding (GloVe) and contextual (GPT-2 induced) embedding methods. The findings suggest an enhanced decoding performance when using contextual encoding at numerous time-points (FDR corrected). This analysis was conducted by employing a paired-sample t-test at each lag, during which the AUCs of all labels were compared separately for each individual lag. All shaded lines represent standard error above and standard error below average. In purple asterisks, we mark the significant difference, one-sided p-value ($p<0.001$), between the average ROC-AUC scores ($n=1100$) based on the IFG and precentral embeddings, using paired sample permutation and Bonferroni correction for multiple comparisons.

Supplementary Information - Decoding architecture

reshape (Reshape)	(None, 10, 45)	0
conv1d (Conv1D)	(None, 8, 128)	
activation (Activation)	(None, 8, 128)	0
batch_normalization(BatchNorm)	(None, 8, 128)	512
dropout (Dropout)	(None, 8, 128)	0
max_pooling1d (MaxPooling1D	(None, 4, 128)	0
conv1d_1 (Conv1D)	(None, 3, 128)	32768
activation_1 (Activation)	(None, 3, 128)	0
batch_normalization_1 (BatchNorm)	(None, 3, 128)	512
dropout_1 (Dropout)	(None, 3, 128)	0
locally_connected1d (Locally connect.)	(None, 2, 128)	65792
batch_normalization_2 (BatchNorm	(None, 2, 128)	512
activation_2 (Activation)	(None, 2, 128)	0
global_max_pooling1d	(None, 128)	0
dense_to_vec (Dense)	(None, 1600)	
layer_normalization (LayerNorm	(None, 1600)	3200

===

Total params: 326,976

Trainable params: 326,208

Non-trainable params: 768

batch size: 32

epochs: 1000

patience: 150

lr: 5e-5

reg conv: 0.35

reg dense: 0.05

dropout: 20%

Table 1

Symbolic Feature	Feature Dimension	Feature Categories / Binary Columns
Part of Speech (POS)	11	NOUN, VERB, DET, ADP, PRON, ADJ, ADV, CONJ, PRT, NUM, X
Stop Word	1	is_stop_word
Shape	16	xxxx, xxx, xx, Xxxxx, Xxx, x, X, Xxxx, Xx, XXXX, XxxXxxxx, XxxxxXx, XxxxxX, XxxxX, Xd, XX
Prefix	19	re, in, de, pro, pre, dis, im, inter, un, up, out, down, non, over, mid, super, trans, auto, mis
Suffix	28	NOUN: al, er, tion, ment, or, ty, ry, age, ity, ee, ance, ist, sion, ship, dom<br>VERB: en, ate, ize<br>ADJ: y, ic, able, ous, ence, ive, ish, ful, ese<br>ADV: ly

Table 1 Symbolic features included in the symbolic embedding.
