# Supplementary material for: Alignment of brain embeddings and artificial contextual embeddings in natural language points to common geometric patterns
Source: Nat Commun. 2024 Mar 30;15:2768. doi: 10.1038/s41467-024-46631-y (PMC10980748; doi:10.1038/s41467-024-46631-y)
Supplement: Supplementary file 2 — Reporting Summary [file 41467_2024_46631_MOESM2_ESM.pdf]

Corresponding author(s): Ariel Goldstein

Last updated by author(s): 2023-12-6

Reporting Summary

Nature Portfolio wishes to improve the reproducibility of the work that we publish. This form provides structure for consistency and transparency in reporting. For further information on Nature Portfolio policies, see our Editorial Policies and the Editorial Policy Checklist.

Statistics

For all statistical analyses, confirm that the following items are present in the figure legend, table legend, main text, or Methods section.

n/a	Confirmed	
☐	☒	The exact sample size (*n*) for each experimental group/condition, given as a discrete number and unit of measurement
☐	☒	A statement on whether measurements were taken from distinct samples or whether the same sample was measured repeatedly
☐	☒	The statistical test(s) used AND whether they are one- or two-sided<br>*Only common tests should be described solely by name; describe more complex techniques in the Methods section.*
☒	☐	A description of all covariates tested
☐	☒	A description of any assumptions or corrections, such as tests of normality and adjustment for multiple comparisons
☐	☒	A full description of the statistical parameters including central tendency (e.g. means) or other basic estimates (e.g. regression coefficient) AND variation (e.g. standard deviation) or associated estimates of uncertainty (e.g. confidence intervals)
☐	☒	For null hypothesis testing, the test statistic (e.g. *F*, *t*, *r*) with confidence intervals, effect sizes, degrees of freedom and *P* value noted<br>*Give P values as exact values whenever suitable.*
☒	☐	For Bayesian analysis, information on the choice of priors and Markov chain Monte Carlo settings
☒	☐	For hierarchical and complex designs, identification of the appropriate level for tests and full reporting of outcomes
☒	☐	Estimates of effect sizes (e.g. Cohen's *d*, Pearson's *r*), indicating how they were calculated

Our web collection on statistics for biologists contains articles on many of the points above.

Software and code

Policy information about availability of computer code

Data collection	Recordings from grid, strip and depth electrode arrays were acquired using one of two amplifier types: NicoletOne C64 clinical amplifier (Natus Neurologics), band-pass filtered from 0.16–250 Hz, and digitized at 512 Hz; NeuroWorks Quantum Amplifier recorded at 2,048 Hz, high-pass filtered at 0.01 Hz and then resampled to 512 Hz.
Data analysis	Data were preprocessed using Matlab 2019b and The Fieldtrip toolbox. Data was analyzed using python packages are specified here https://github.com/hassonlab/247-main/blob/main/env.yml. Brain plots were done using toolbox for MATLAB available at (https://github.com/HughWXY/ntools_elec). All scripts for analyses are available at:All the scripts for analyses can be found at: https://github.com/orgs/hassonlab/repositories. analyses were done using python 3.8.

For manuscripts utilizing custom algorithms or software that are central to the research but not yet described in published literature, software must be made available to editors and reviewers. We strongly encourage code deposition in a community repository (e.g. GitHub). See the Nature Portfolio guidelines for submitting code & software for further information.

Data

Policy information about availability of data

All manuscripts must include a data availability statement. This statement should provide the following information, where applicable:

- Accession codes, unique identifiers, or web links for publicly available datasets
- A description of any restrictions on data availability
- For clinical datasets or third party data, please ensure that the statement adheres to our policy

The neural data are available under restricted access for it may contain sensitive information, access can be obtained by thus we establishing collaboration with the authors.

Research involving human participants, their data, or biological material

Policy information about studies with human participants or human data. See also policy information about sex, gender (identity/presentation), and sexual orientation and race, ethnicity and racism.

Reporting on sex and gender	Three patients (2 females (gender assigned based on medical record); 24–48 years old)<br>Sex or gender are not relevant variables to this study, and analysis was not done considering it.
Reporting on race, ethnicity, or other socially relevant groupings	Three patients (2 females (gender assigned based on medical record); 24–48 years old)
Population characteristics	All the patients suffer from drug immune epilepsy. We select patients with intact cognitive faculty as determined by neuropsychological tests that are done as part of the hospitalization process.
Recruitment	. Each participant provided informed consent following protocols approved by the New York University Grossman School of Medicine Institutional Review Board. Patients were informed that participation in the study was unrelated to their clinical care and that they could withdraw from the study without affecting their medical treatment. As the patients volunteer to the experiment there is a potential to self-selection based on this trait. However, the researcher could not think how it could impact the results.
Ethics oversight	Ethical oversight<br>Princeton University and New York University School of Medicine respective Institutional Review Boards approved the Studies.<br>. Each participant provided informed consent following protocols approved by the New York University Grossman School of Medicine Institutional Review Board. Patients were informed that participation in the study was unrelated to their clinical care and that they could withdraw from the study without affecting their medical treatment.

Note that full information on the approval of the study protocol must also be provided in the manuscript.

Field-specific reporting

Please select the one below that is the best fit for your research. If you are not sure, read the appropriate sections before making your selection.

☐ Life sciences ☒ Behavioural & social sciences ☐ Ecological, evolutionary & environmental sciences

For a reference copy of the document with all sections, see nature.com/documents/nr-reporting-summary-flat.pdf

Behavioural & social sciences study design

All studies must disclose on these points even when the disclosure is negative.

Study description	we use densely recorded neural activity patterns in the inferior frontal gyrus (IFG) of three participants using dense intracranial arrays while they listened to a 30-minute podcast. From these fine-grained spatiotemporal neural recordings, we derive a continuous vectorial representation for each word (i.e., a brain embedding) in each patient. We dcorrelated brain embeddings in the IFG and the DLM contextual embedding induced by GPT-2 at different time points.
Research sample	The research sample consists of three patients with dense recordings of their IFG. The dense grid research technology is only employed by a few groups world wide, especially chronically, we believe that in the future more of this type of data will be available. Gender is assigned based on medical reports (we now specify in text). We did not analyze the effects per sex as the sample size is small and that we replicated the effect per participant.

Sampling strategy	Three patients (2 females (gender assigned based on medical record); 24–48 years old) with treatment-resistant epilepsy undergoing intracranial monitoring with subdural grid and strip electrodes for clinical purposes participated in the study. No statistical method was used to predetermine sample size.
Data collection	Recordings from grid, strip and depth electrode arrays were acquired using one of two amplifier types: NicoletOne C64 clinical amplifier (Natus Neurologics), band-pass filtered from 0.16–250 Hz, and digitized at 512 Hz; NeuroWorks Quantum Amplifier recorded at 2,048 Hz, high-pass filtered at 0.01 Hz and then resampled to 512 Hz. 570 electrodes were placed on the left hemisphere. 18 electrodes from all patients were removed due to faulty recordings.<br><br>The participants as well as the personal who interacted with the patients were not aware of the study hypothesis.
Timing	Each patient cam to the hospital at 2019. The recording was continous 30 min. The exact date of collection is an identifiable information.
Data exclusions	18 electrodes due to faulty recordings
Non-participation	No patients were removed.
Randomization	As analyses can be seen for each participant (seperatly and together) there is no between particiapant analysis nor allocation to conditions.

Reporting for specific materials, systems and methods

We require information from authors about some types of materials, experimental systems and methods used in many studies. Here, indicate whether each material, system or method listed is relevant to your study. If you are not sure if a list item applies to your research, read the appropriate section before selecting a response.

Materials & experimental systems

n/a	Involved in the study	
☒	☐	Antibodies
☒	☐	Eukaryotic cell lines
☒	☐	Palaeontology and archaeology
☒	☐	Animals and other organisms
☒	☐	Clinical data
☒	☐	Dual use research of concern
☒	☐	Plants

Methods

n/a	Involved in the study	
☒	☐	ChIP-seq
☒	☐	Flow cytometry
☒	☐	MRI-based neuroimaging
